# Supplementary material for: Engineered MED12 mutations drive leiomyoma-like transcriptional and metabolic programs by altering the 3D genome compartmentalization
Source: Nat Commun. 2023 Jul 10;14:4057. doi: 10.1038/s41467-023-39684-y (PMC10333368; doi:10.1038/s41467-023-39684-y)
Supplement: Supplementary file 1 — Supplementary Information [file 41467_2023_39684_MOESM1_ESM.pdf]

Supplementary Figures: Buyukcelebi et al.

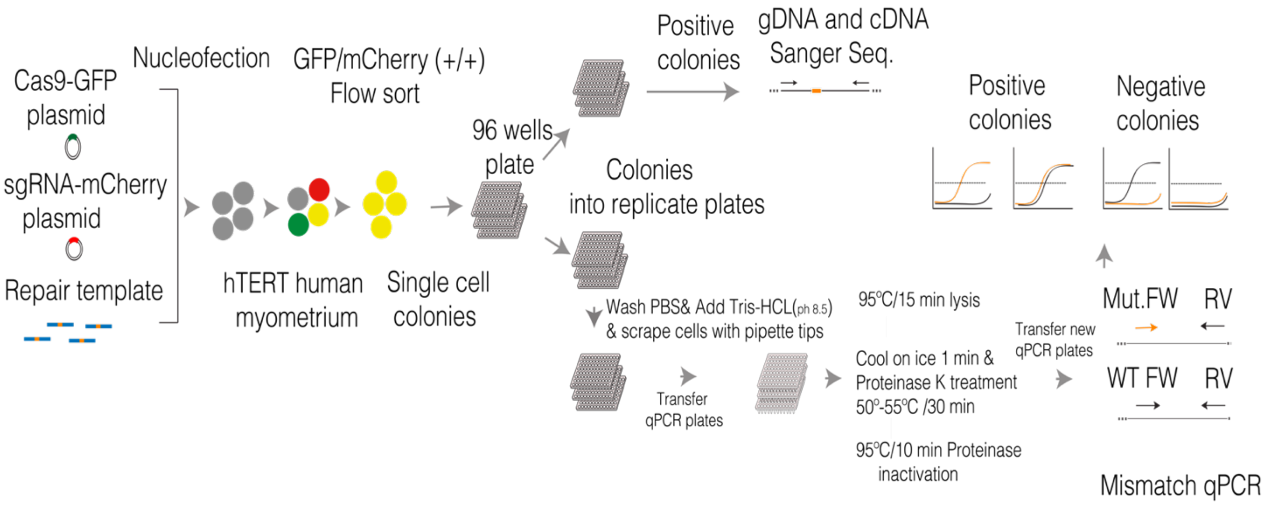

Supplementary Figure 1: General strategy for engineering MED12- Gly 44 mutation in hTERT cells through CRISPR-based knock-in and qPCR-based single-cell colony selection. hTERT cells were nucleofected with MED12 exon 2 targeting sgRNA (mCherry), Cas9-GFP, and single strand HDR template (50 pmol) using Neon transfection system as set up parameters 1400 Voltage/ 20 with/ 2 pulse. 48 hr later, double positive (mcherry+GFP) cells were selected using FACS and seeded as single cells in 96 well plates. Single-cell colonies were split into replicate plates. After colonies reached a certain size, cells in one of the replicate plates were lysed in the plate and were transferred to a qPCR plate for PCR-based colony screening on the genomic DNA. After detecting mutation-positive colonies, they were selected in the replicate plates, and mutation was validated by gDNA and cDNA Sanger sequencing.

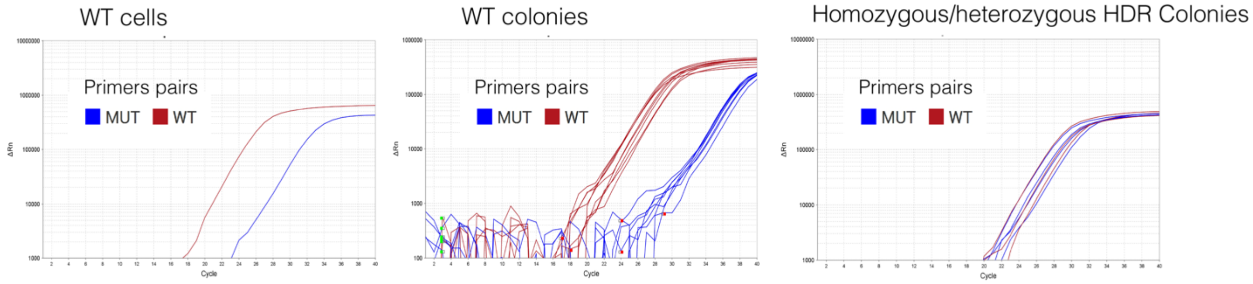

Supplementary Figure 2: qPCR-based colony screening. qPCR amplification plots show DNA content after amplification of DNA from WT template or single cell colonies with WT-specific and MED12-mutations-specific PCR primers. WT-Forward and Reverse primer pair amplifies homozygous WT genomic DNA earlier than MUT-Forward and Reverse primer pair, but in MED12 homozygous or heterozygous mutant cells, the MUT-F and R primer amplify genomic DNA at the same or better rate than WT-Forward-reverse primer pairs.

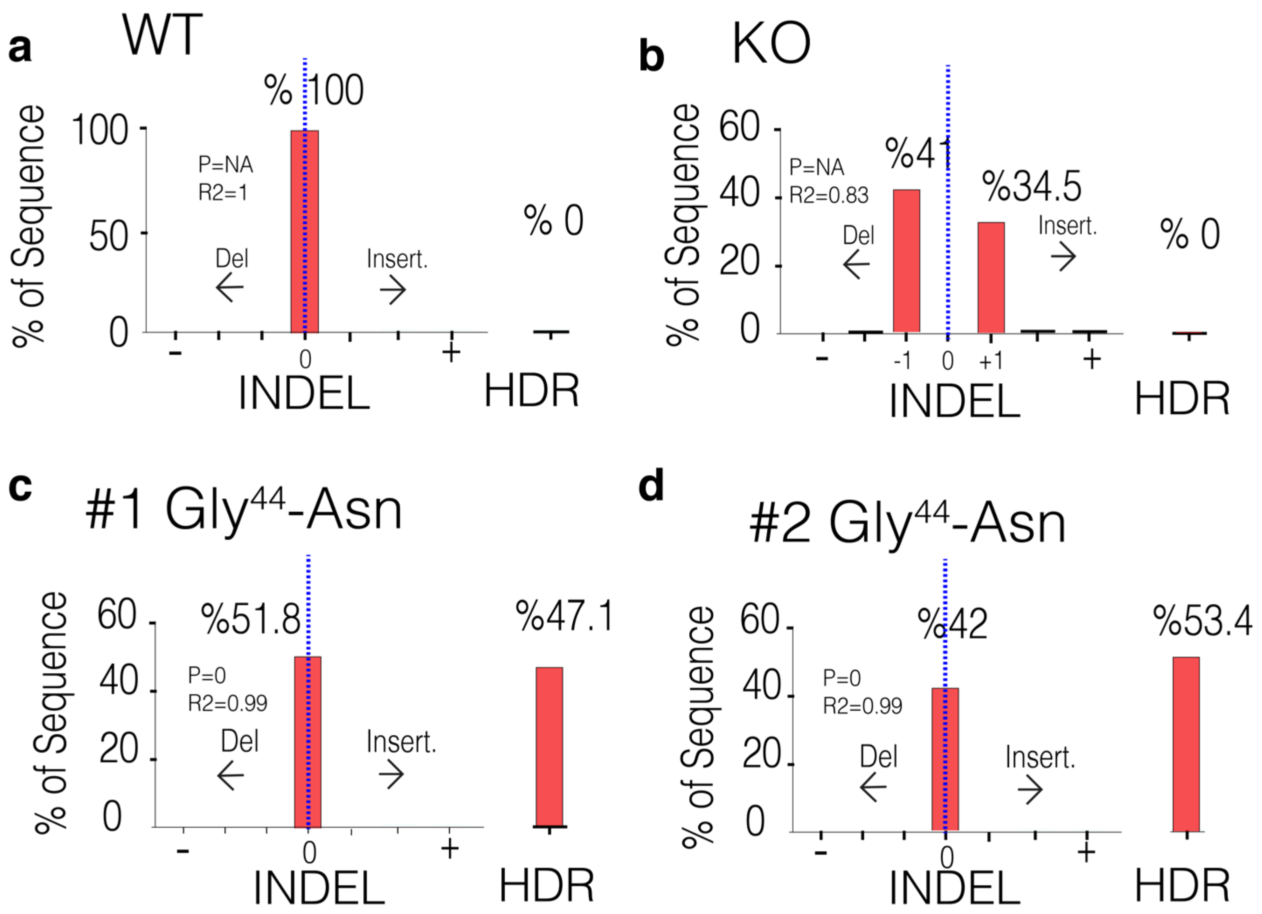

Supplementary Figure 3: CRISPR-TIDER analysis of genomic DNA of single-cell colonies. Genomic DNA from WT cells (a), MED12-KO single-cell colony (b), and two separate MED12-Gly 44 mutant single-cell colonies (c-d) were Sanger sequenced, and the resulting chromatograms were processed for CRISPR-TIDER analysis. WT cells have %100 WT alleles (0 indels) for the MED12 exon2 sequence, whereas the MED12-KO cells has 1 nucleotide deletion in one allele and 1 nucleotide insertion in the other allele. MED12 exon2 region sequence of mutant colonies has MED12-Gly-44 mutation and WT-MED12 exon2 sequence.

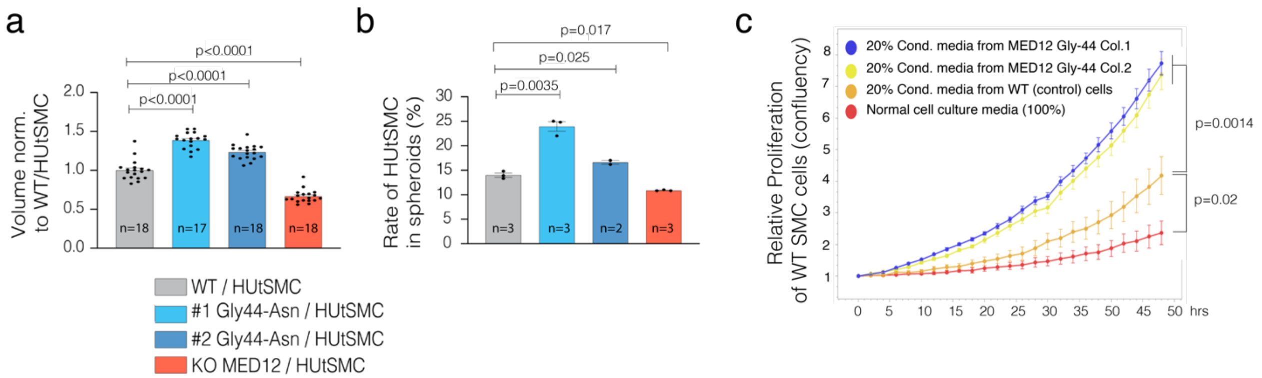

Supplementary Figure 4: MED12 mutant cells increased proliferation rate of non-mutant smooth muscle cells in 3D condition. a) The same number of MED12 mutant and mCherry-labeled primary Human Uterine Smooth Muscle Cells (HUtSMC) cells (1500/1500 cells) were counted and seeded at a low attachment plate. After four days, the spheroid photos were taken by the EVOS cell imaging system. All spheroid volumes normalized mean WT/HUtSMC spheroid volume. **b)** The rate of proliferation in non-mutant HUtSMC cells in spheroids was calculated by counting the mCherry-positive cells/all cells by flow cytometry. **c)** The Incusyte experiment indicates the effect of condition media (20%) from MED12 mutant and WT cells on the proliferation of WT SMC. For statistical significance, a two-tailed unpaired t-test has been used.

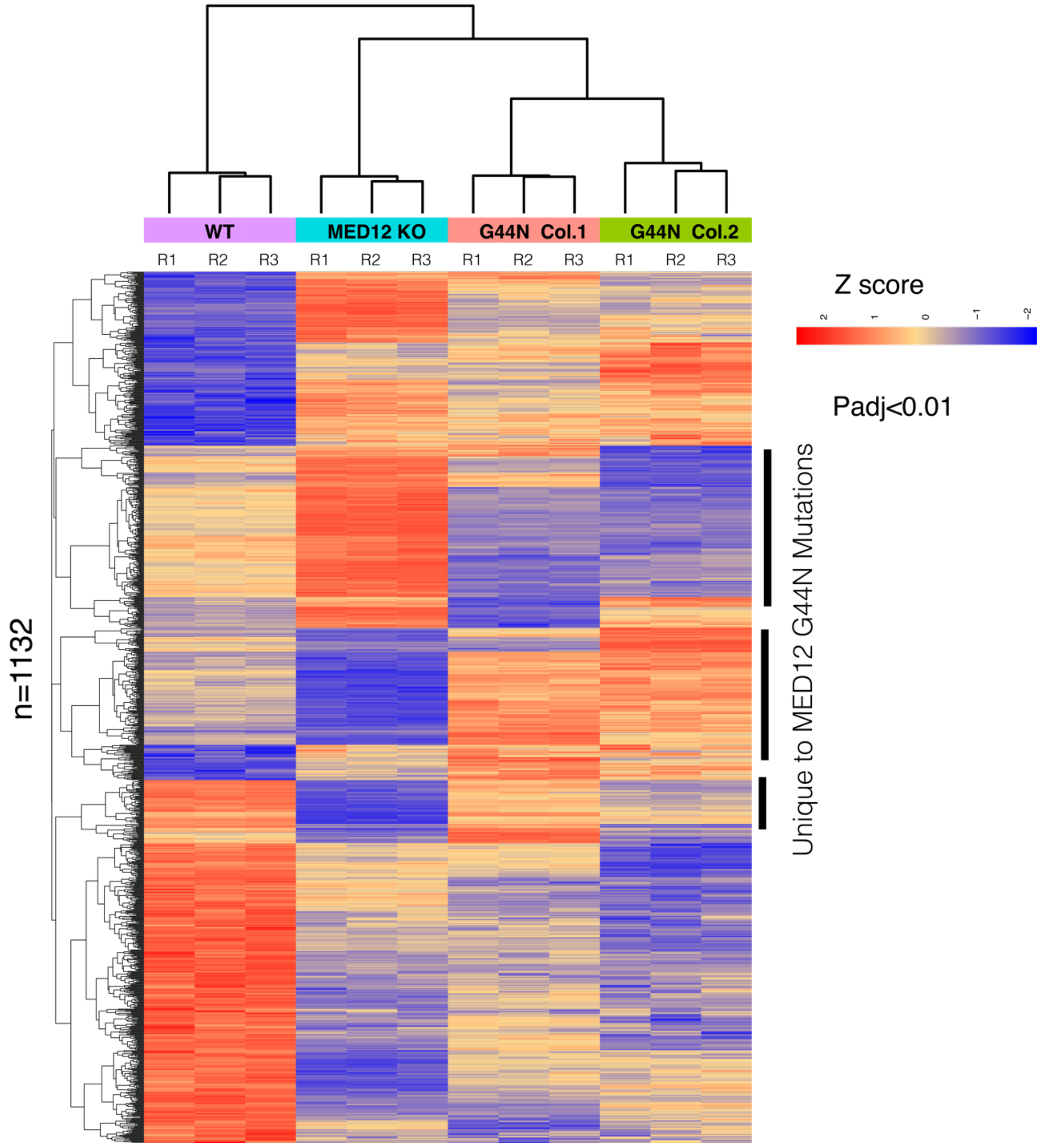

Supplementary Figure 5: The MED12 Gly44 mutant cells have distinct transcriptional states than WT or MED12 KO cells. The heatmap shows differentially expressed genes (padj<0.01) in MED12 WT, KO, and MED12 Gly44 mutant cell colonies (Mut-1 and Mut-2). Heatmap was drawn based on the normalized z-score. The gene names and z scores are presented in **Supplementary Data 3**.

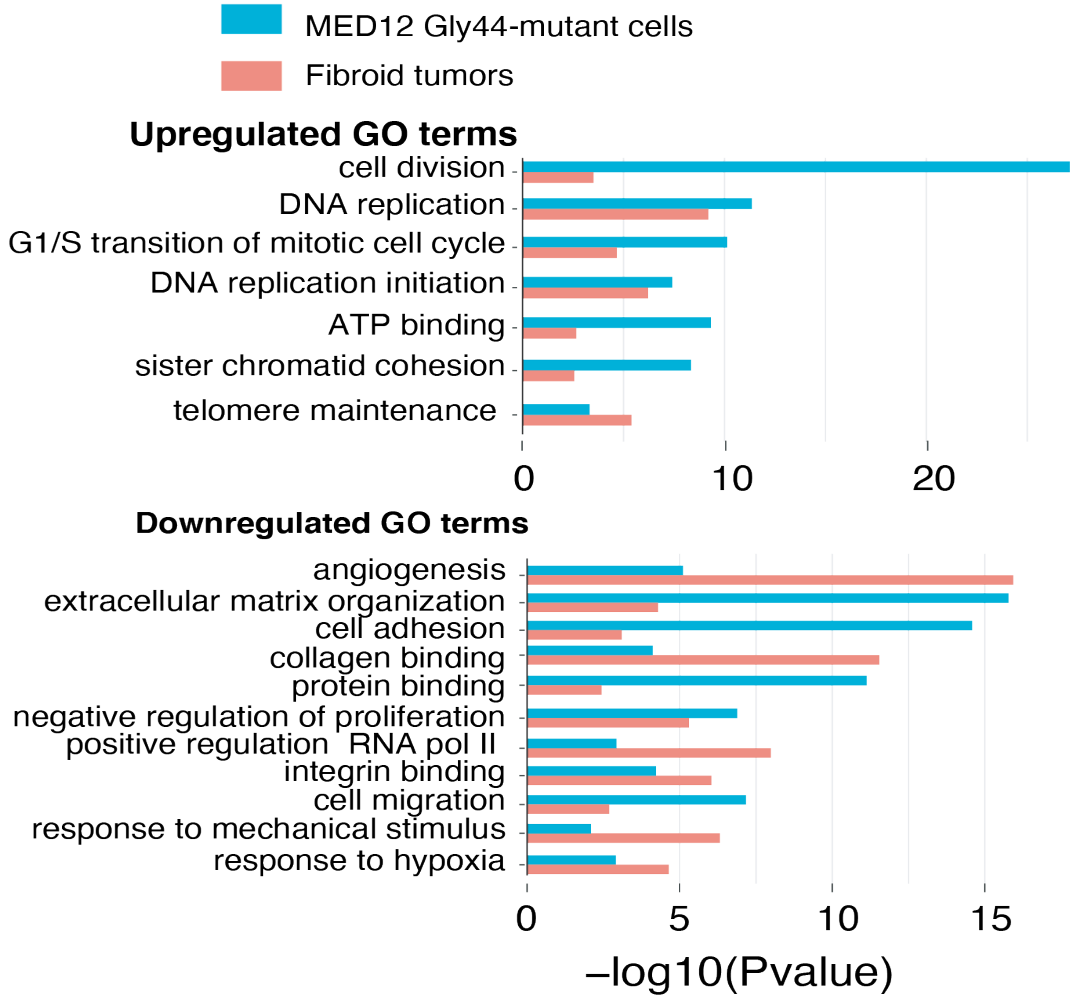

Supplementary Figure 6: The gene ontology (GO) analyses on differentially expressed genes in Gly44 mutant cells and fibroid tumors identified a common set of biological processes between these two samples. Bar plots show common gene ontology terms between MED12 mutant cells and fibroid tumors for the differentially upregulated and downregulated genes (p adj<0.01). 671 upregulated genes in MED12 mutant cells, 3079 upregulated genes in leiomyoma, 793 downregulated genes in MED12 mutant cells, and 2752 downregulated genes in leiomyoma were uploaded to DAVID Bioinformatics Resources and gene ontology (GO) term analysis was done with default settings. The top common GO terms in mutant cells and leiomyoma are displayed in horizontal bar plots.

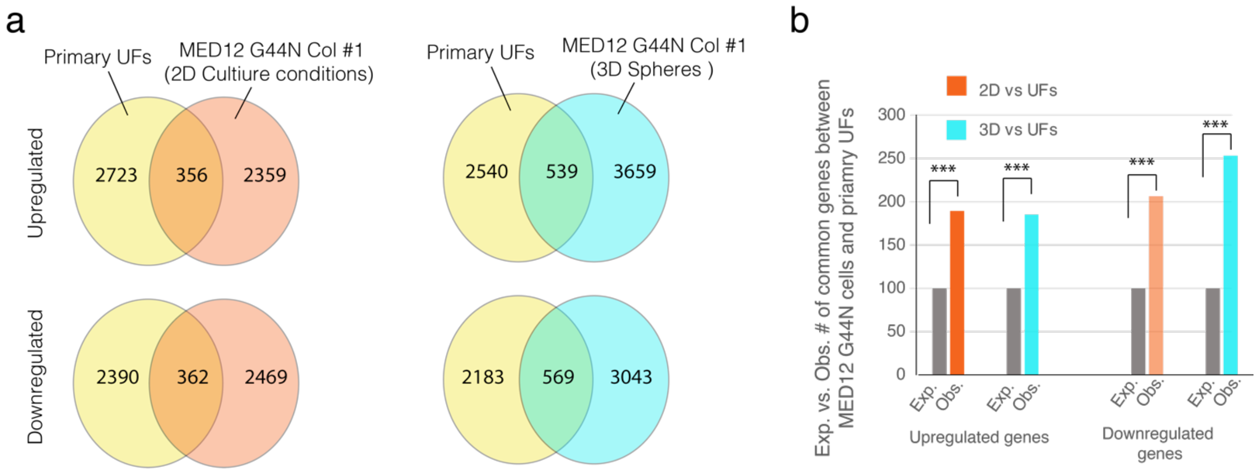

Supplementary Figure 7: a. The Venn diagrams show an overlap between the number of differentially expressed up- and downregulated genes between primary fibroid tumors vs the engineered MED12 mutant cells (colony #1) that were cultured in 2D or 3D culture conditions. Due to substantial sample size difference, the *Padj* value for fibroid samples (n=15) was chosen to be <0.01, but for MED12-Gly-44 mutation the *Padj* value is <0.05. **b.** Observed vs Expected number of common genes under these conditions. *** indicates p<0.001 (hypergeometric test)

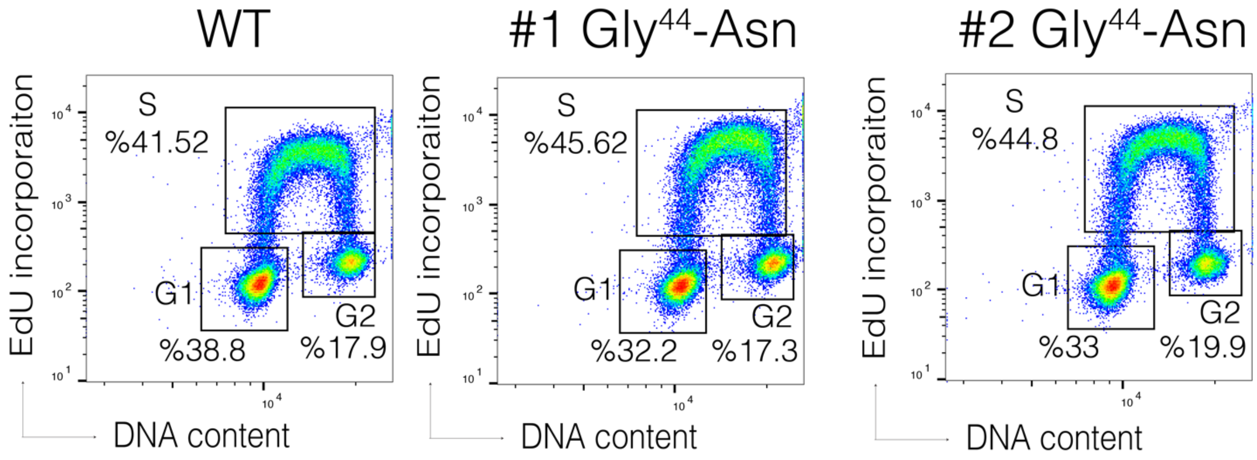

Supplementary Figure 8: Gly44-MED12 mutant cells have a significantly higher percentage of cells in the S-phase. Flow-Jo analysis of flow cytometry result shows EdU incorporation and DNA content in WT and two separate MED12 mutant colonies. Cells were treated 10uM/90 min Edu and stained for DNA content by FxCycle™ Violet Stain. The same gate parameters were used for all samples to detect the cell percentage of the population. 488 nm excitation with a green emission filter was used for the detection of Edu.

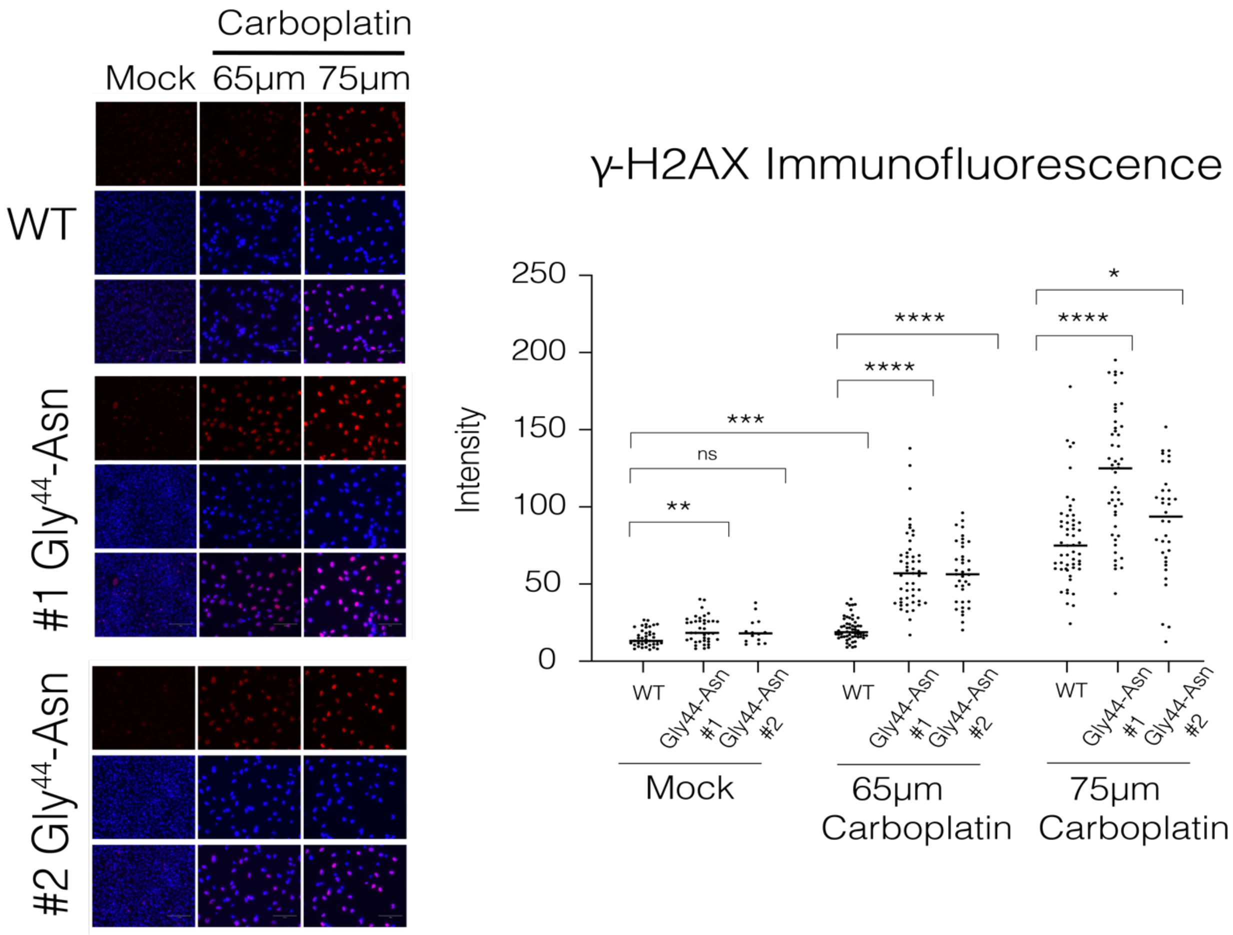

Supplementary Figure 9: Immunofluorescence staining of γH2AX assessing DNA damage in WT and MED12 mutant cells after Carboplatin treatment. Three replicates of cells were seeded in 6 well plates and treated for three days with 75um and 65 um of Carboplatin. And they were stained with DAPI and γH2AX after treatment. The images were analyzed using Fiji software. Dot plots show quantified fluorescent intensity of the randomly selected cells from the pictures (****p<0.0001, ***p<0.001, **p<0.01, *p<0.05, Two-sided unpaired t-test)

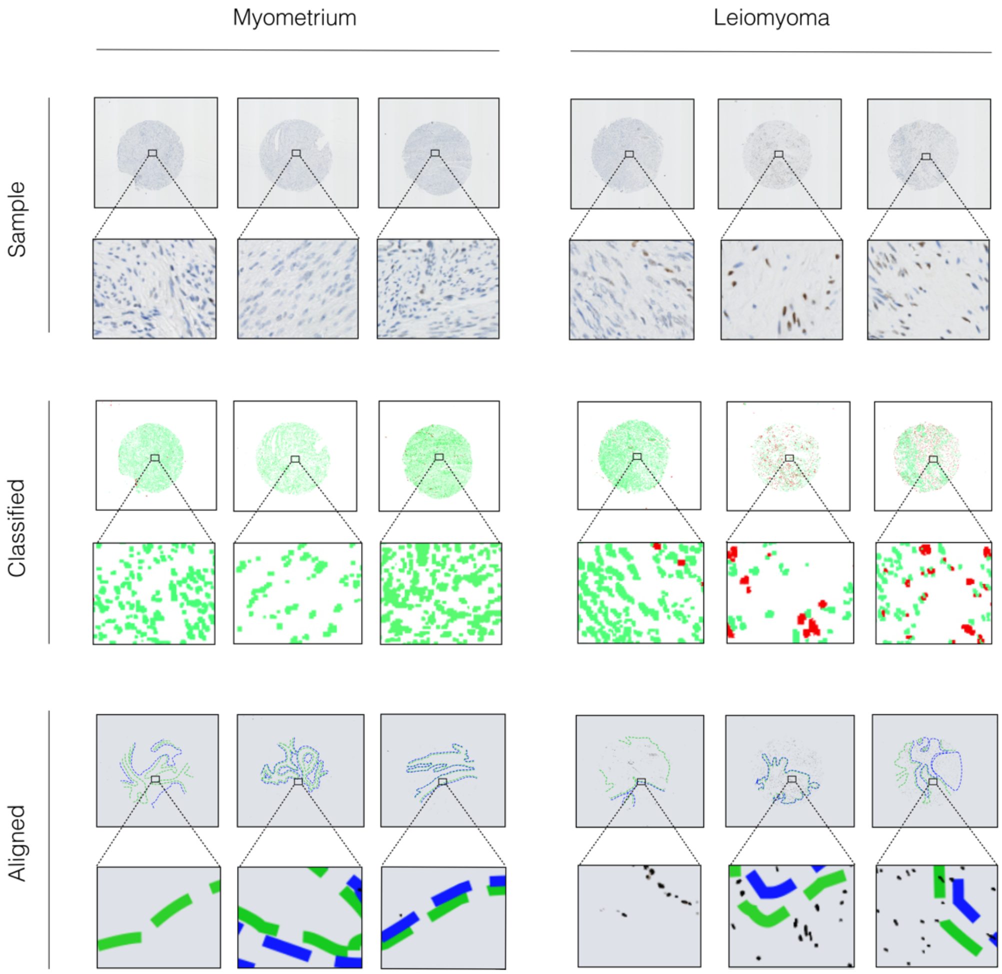

Supplementary Figure 10: An extensive overview of the γH2AX staining analysis. Classified images were generated by machine learning, with green shapes indicating γH2AX undyed nuclei and red shapes indicating γH2AX stained nuclei. The Aligned images were created with the semi-automated approach to assess the power of the machine learning and compare the γH2AX staining distribution between the stromal and muscle tissues of each sample. Green dotted line areas in the aligned images designate muscle tissue and blue dotted line regions to show stromal tissue.

a

	Total pairs	Mapped pairs	Mapping ratio	Nodup pairs	Cis	Cis (>20kb)	Long range ratio	trans	Trans ratio
WT	804,255,654	570,972,381	0.7099	413,678,879	291,668,576	168,379,417	0.4070	122,010,303	0.2949
Mut	717,644,659	512,613,431	0.7143	318,973,324	283,583,474	162,670,211	0.5100	35,389,850	0.1109

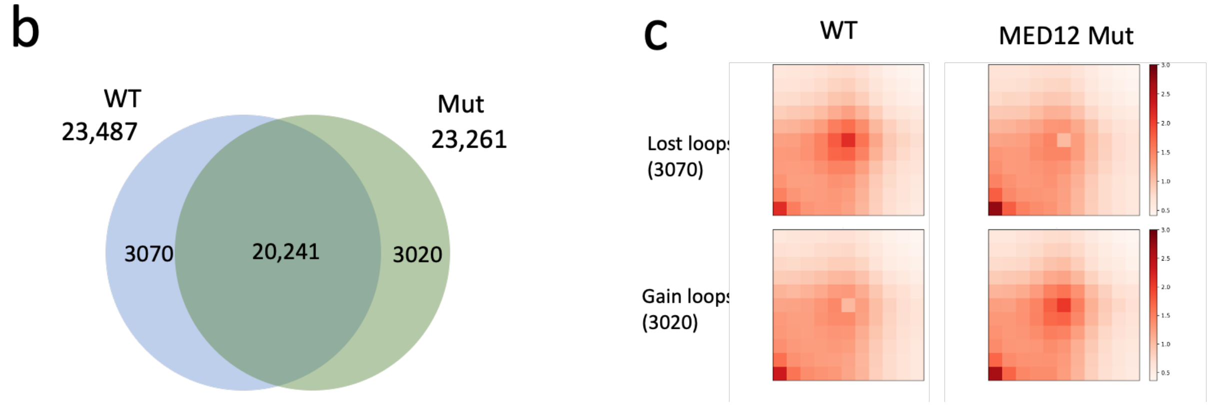

Supplementary Figure 11: MED12 mutations alter the contact frequency for a small set of chromatin loops. a. Summary of mapping statistics of Hi-C chromatin maps in WT and MED12 mutant cells. **b** The Venn diagram shows the overlap of the number of chromatin loops in WT and MED12 mutant cells. **c.** The heatmaps show the Hi-C loop contact frequency of the lost and gained loops in WT and MED12 mutant cells.

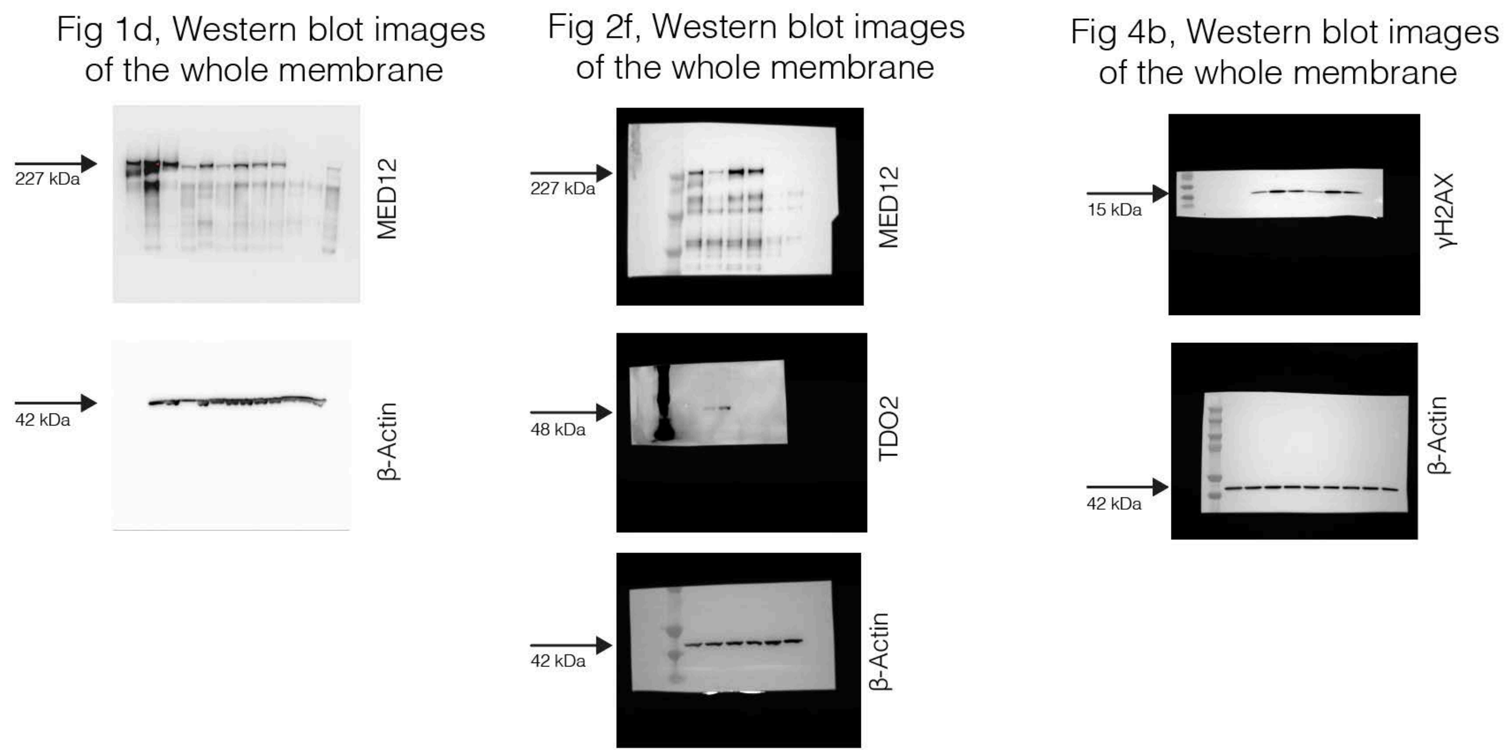

Supplementary Figure 12: Whole membrane western blot images are shown for the processed western blot data presented in **Figure 1d, Figure 2f** and **Figure 4b**
