# Supplementary material for: Engineered MED12 mutations drive leiomyoma-like transcriptional and metabolic programs by altering the 3D genome compartmentalization
Source: Nat Commun. 2023 Jul 10;14:4057. doi: 10.1038/s41467-023-39684-y (PMC10333368; doi:10.1038/s41467-023-39684-y)
Supplement: Supplementary file 2 — Reporting Summary [file 41467_2023_39684_MOESM2_ESM.pdf]

nature portfolio

Corresponding author(s): Mazhar Adli

Last updated by author(s): Jun 23, 2023

Reporting Summary

Nature Portfolio wishes to improve the reproducibility of the work that we publish. This form provides structure for consistency and transparency in reporting. For further information on Nature Portfolio policies, see our Editorial Policies and the Editorial Policy Checklist.

Statistics

For all statistical analyses, confirm that the following items are present in the figure legend, table legend, main text, or Methods section.

n/a	Confirmed	
☐	☒	The exact sample size (n) for each experimental group/condition, given as a discrete number and unit of measurement
☒	☐	A statement on whether measurements were taken from distinct samples or whether the same sample was measured repeatedly
☐	☒	The statistical test(s) used AND whether they are one- or two-sided<br>*Only common tests should be described solely by name; describe more complex techniques in the Methods section.*
☒	☐	A description of all covariates tested
☐	☒	A description of any assumptions or corrections, such as tests of normality and adjustment for multiple comparisons
☒	☐	A full description of the statistical parameters including central tendency (e.g. means) or other basic estimates (e.g. regression coefficient) AND variation (e.g. standard deviation) or associated estimates of uncertainty (e.g. confidence intervals)
☒	☐	For null hypothesis testing, the test statistic (e.g. F, t, r) with confidence intervals, effect sizes, degrees of freedom and P value noted<br>*Give P values as exact values whenever suitable.*
☒	☐	For Bayesian analysis, information on the choice of priors and Markov chain Monte Carlo settings
☒	☐	For hierarchical and complex designs, identification of the appropriate level for tests and full reporting of outcomes
☒	☐	Estimates of effect sizes (e.g. Cohen's d, Pearson's r), indicating how they were calculated

Our web collection on statistics for biologists contains articles on many of the points above.

Software and code

Policy information about availability of computer code

Data collection	Not applicable
Data analysis	No custom software or algorithm was used in this study. All software used in this study, including version numbers, are also described in the methods. Software includes: CRISPOR (available online), Incucyte cell-by-cell analysis,FlowJo,Picard tool, bedtools, MACS2,CRISPR-TIDER, STAR aligner, Bowtie, FastQC, featureCounts,DeepTools, Prism-GraphPad, Metaboanalyst (available online), runHIC, Trim Galore, Burrows-Wheeler Aligner, cooltools, Fiji<br>R packages used in this study include DESeq2, DiffBind, pheatmaps, ggplot2,ChipSeeker

For manuscripts utilizing custom algorithms or software that are central to the research but not yet described in published literature, software must be made available to editors and reviewers. We strongly encourage code deposition in a community repository (e.g. GitHub). See the Nature Portfolio guidelines for submitting code & software for further information.

Data

Policy information about availability of data

All manuscripts must include a data availability statement. This statement should provide the following information, where applicable:

- Accession codes, unique identifiers, or web links for publicly available datasets
- A description of any restrictions on data availability
- For clinical datasets or third party data, please ensure that the statement adheres to our policy

All metabolomics and high throughput sequencing data as well as gene expression data will be made publicly available.

Human research participants

Policy information about studies involving human research participants and Sex and Gender in Research.

Reporting on sex and gender	*Use the terms sex (biological attribute) and gender (shaped by social and cultural circumstances) carefully in order to avoid confusing both terms. Indicate if findings apply to only one sex or gender; describe whether sex and gender were considered in study design whether sex and/or gender was determined based on self-reporting or assigned and methods used. Provide in the source data disaggregated sex and gender data where this information has been collected, and consent has been obtained for sharing of individual-level data; provide overall numbers in this Reporting Summary. Please state if this information has not been collected. Report sex- and gender-based analyses where performed, justify reasons for lack of sex- and gender-based analysis.*
Population characteristics	*Describe the covariate-relevant population characteristics of the human research participants (e.g. age, genotypic information, past and current diagnosis and treatment categories). If you filled out the behavioural & social sciences study design questions and have nothing to add here, write "See above."*
Recruitment	*Describe how participants were recruited. Outline any potential self-selection bias or other biases that may be present and how these are likely to impact results.*
Ethics oversight	*Identify the organization(s) that approved the study protocol.*

Note that full information on the approval of the study protocol must also be provided in the manuscript.

Field-specific reporting

Please select the one below that is the best fit for your research. If you are not sure, read the appropriate sections before making your selection.

☒ Life sciences ☐ Behavioural & social sciences ☐ Ecological, evolutionary & environmental sciences

For a reference copy of the document with all sections, see nature.com/documents/nr-reporting-summary-flat.pdf

Life sciences study design

All studies must disclose on these points even when the disclosure is negative.

Sample size	No statistical methods were used to determine sample sizes.
Data exclusions	No data were excluded from the analysis.
Replication	Biological replicates (MED12-Gly44 mutant cells etc.) were processed in separate batches and the experimental procedure for each experiment type (RNA-seq/ Cut-tag/ HI-C/LC-MS) was repeated on separate occasions for each batch to ensure reproducibility of the experimental protocol.
Randomization	Due to the experimental design and goals of this study, all MED12-Gly44 mutant hTERT cells were included in this study.
Blinding	Due to the experimental design in which cells had to be confirmed to be normal (WT) or MED12 mutant , blinding was not possible during sample preparation or during data collection and analysis.

Reporting for specific materials, systems and methods

We require information from authors about some types of materials, experimental systems and methods used in many studies. Here, indicate whether each material, system or method listed is relevant to your study. If you are not sure if a list item applies to your research, read the appropriate section before selecting a response.

Materials & experimental systems

n/a	Involved in the study
☐	☒ Antibodies
☐	☒ Eukaryotic cell lines
☒	☐ Palaeontology and archaeology
☒	☐ Animals and other organisms
☒	☐ Clinical data
☒	☐ Dual use research of concern

Methods

n/a	Involved in the study
☐	☒ ChIP-seq
☐	☒ Flow cytometry
☒	☐ MRI-based neuroimaging

Antibodies

Antibodies used

All antibodies used in this study and their catalog numbers are also listed in the methods.
Cut&tag antibodies: H3K27ac antibody (ab4729, Abcam)
Western blot antibodies: MED12(Bethyl lab, #A300-774A), TDO2 (Protein-tech,#15880-1AP), Phospho-Histone H2A.X (Ser139)(Cell Signaling,#2577), Anti-β-Actin antibody (Sigma, Mouse monoclonal, #A1978-100UL)
γH2AX staining of TMA: γH2AX (Phospho-Histone H2A.X (Ser139) (20E3) Rabbit mAb (Cell signaling, #9718))
Immunofluorescence staining: γH2AX (Phospho-Histone H2A.X (Ser139)(Cell Signaling,#2577))

Validation

- H3K27Ac antibody was validated by the manufacturer and independently validated for specificity: http://www.histoneantibodies.com/FinalArrayData/H3K27ac/
- MED12 antibody was validated by independently for specificity within our lab group by western blot of MED12-KO cells.
- MED12,TDO2,Phospho-Histone H2A.X ,Anti-β-Actin antibody have been used in previously published western blot studies and were also validated by the manufacturer.

Eukaryotic cell lines

Policy information about cell lines and Sex and Gender in Research

Cell line source(s)

Human myometrial cell line myo-hTERT was kindly provided by C. Mendelson (UT Southwestern)
Human uterine smooth muscle primary cells (HUtSMC). Obtained from American Type Culture Collection (ATCC, cat # PCS-460-011)

Authentication

Immortalized myometrial cells obtained from uterus (Condon et al., 2002).
New vial of authenticated primary cells were obtained directly from American Type Culture Collection (ATCC, cat # PCS-460-011).

Mycoplasma contamination

Human myometrial cell line myo-hTERT was confrmed as mycoplasma-free using ATCC UNIVERSAL MYCOPLASMA DETECTION #301012K)
New vial of mycoplasma-free primary cells were obtained directly from American Type Culture Collection (ATCC, cat # PCS-460-011).

Commonly misidentified lines
(See ICLAC register)

No commonly misidentified cell lines were used in this study.

ChIP-seq

Data deposition

☐ Confirm that both raw and final processed data have been deposited in a public database such as GEO.

☐ Confirm that you have deposited or provided access to graph files (e.g. BED files) for the called peaks.

Data access links
May remain private before publication.

For "Initial submission" or "Revised version" documents, provide reviewer access links. For your "Final submission" document, provide a link to the deposited data.

Files in database submission

Provide a list of all files available in the database submission.

Genome browser session
(e.g. UCSC)

Provide a link to an anonymized genome browser session for "Initial submission" and "Revised version" documents only, to enable peer review. Write "no longer applicable" for "Final submission" documents.

Methodology

Replicates

Duplicate

Sequencing depth

Sequencing depth of all experiments will be provided in the supplementary materials.

Antibodies	H3K27ac antibody (ab4729, Abcam)
Peak calling parameters	Data analysis and peak calling parameters are provided in the methods.
Data quality	Reads quality was checked using FASTQc .All peaks were determined at FDR<0.05 using MACS2 package. All peak numbers will be provided in the supplementary materials.
Software	All Cut&tag data analysis software and methodology are described in the methods.

Flow Cytometry

Plots

Confirm that:

- [x] The axis labels state the marker and fluorochrome used (e.g. CD4-FITC).
- [x] The axis scales are clearly visible. Include numbers along axes only for bottom left plot of group (a 'group' is an analysis of identical markers).
- [x] All plots are contour plots with outliers or pseudocolor plots.
- [x] A numerical value for number of cells or percentage (with statistics) is provided.

Methodology

Sample preparation	Sample preparation is described in material and method.
Instrument	BD FACSMelody Cell Sorter (3-laser 10-parameter)
Software	Cells were analyzed using flow cytometry, and the results were analyzed using FlowJo.
Cell population abundance	Douple positive (mCherry/GFP) cell population rate after nucleofection is 2.52%.<br>The abundance of the relevant cell populations within different cell cycle was reported on main figures and supplementary figures.
Gating strategy	488 nm excitation with a green emission filter was used for the detection of Edu.<br>We gated based on the “unstained” sample which is negative for both of the fluorophores. We also included “single-negative samples “ which are negative for either GFP or mCherry fluorophores.

- [ ] Tick this box to confirm that a figure exemplifying the gating strategy is provided in the Supplementary Information.
