# Supplementary material for: Engineered MED12 mutations drive leiomyoma-like transcriptional and metabolic programs by altering the 3D genome compartmentalization
Source: Nat Commun. 2023 Jul 10;14:4057. doi: 10.1038/s41467-023-39684-y (PMC10333368; doi:10.1038/s41467-023-39684-y)
Supplement: Supplementary file 7 — Description of Additional Supplementary Files [file 41467_2023_39684_MOESM7_ESM.pdf]

Title: Supplementary Data 1
Description: Mass-spectrometry quantified metabolite levels in WT and MED12 G44N mutant cells.

Title: Supplementary Data 2
Description: Differentially expressed genes in MED12 Gly44N colonies vs WT (DESEQ, padj<0.05) (This table has been made by DESEQ2 Package)

Title: Supplementary Data 3
Description: Differentially expressed genes in Supplementary Figure 5 (Z score)

Title: Supplementary Data 4
Description: PCR Primers and Oligos
